# Defining an enabling environment for those with chronic disease: an integrative review

**DOI:** 10.1186/s12912-021-00741-w

**Published:** 2021-12-20

**Authors:** Valérie Loizeau, Jean-Manuel Morvillers, Dominique Pougheon Bertrand, Kelley Kilpatrick, Monique Rothan-Tondeur

**Affiliations:** 1 Centre Hospitalier Intercommunal Poissy Saint Germain, Université Sorbonne Paris Nord, Nursing Sciences Research Chair, Laboratory Educations and Health Practices (LEPS), (EA 3412), UFR SMBH, F-93017 Bobigny, France; 2 Research in Nursing Sciences, Health Education and Practice Laboratory (LEPS), (EA 3412), UFR SMBH, F-93017 Bobigny, France; 3 Laboratoire Enseignements et Pratiques de Santé (LEPS), (EA 3412), UFR SMBH, F-93017 Bobigny, France; 4 Susan E. French Chair in Nursing Research and Innovative Practice, Ingram School of Nursing, Faculty of Medicine and Health Sciences, McGill University, 680 Sherbrooke Street West, Office 1811, Montreal, Quebec H3A 2M7 Canada; 5 Research in Nursing Sciences Tondeur, Paris, France; 6 Laboratoire d’Enseignements et de Pratiques de Santé (LEPS), (EA 3412), UFR SMBH, F-93017 Bobigny, France

**Keywords:** Chronic illness, Integrative review, Nurse-patient relationship, Nurse roles, Patient participation, Quality of Life.

## Abstract

**Background:**

Health policies are currently being implemented to cope with the 37% of those affected by chronic disease and 63% of deaths worldwide. Among the proposals, there is accelerating support for greater autonomy for patients, which incorporates several concepts, including empowerment. To achieve this, develop an environment to increase an individual’s capacity for action seems to be a fundamental step. The aim of this study is to characterize an enabling environment for patients in the context of chronic disease management.

**Methods:**

An integrative review design was applied. Medline, CINAHL, and Web of Science databases were searched to identify relevant literature published between 2009 and 2019. Overall, the review process was guided by the PRISMA 2020 checklist. The Mixed Methods Appraisal Tool for quality evaluation was used.

**Results:**

A total of 40 articles were analyzed, divided into 18 quantitative studies, 11 qualitative studies, two mixed studies, seven expert opinions, one theory and one conference report. The following characteristics defining an enabling environment were taken from the literature relating to those with a chronic condition: Needs assessment-adaptation of responses, supporting “take care”, involvement in support, knowledge improvement, engagement with professionals, use of information and communication technologies, and organization of care. Beyond that, the interactions highlighted between these seven categories characterize an enabling environment.

**Conclusion:**

This review specifies the essential elements of an enabling environment for patients with chronic conditions. It encompasses the partnership between the healthcare professional, such as the advanced practice nurse, and the individual for whom interventions and care strategies must be devised.

## Introduction

Chronic diseases, defined by the World Health Organization as non-communicable diseases [1] generate a transition to acute illness [2] and thus represent one of the main global challenges of the twenty-first century [3]. Chronic diseases are responsible for 63% of deaths worldwide, 29% of which are in people under the age of 60 [1], and are therefore the leading cause of death globally. The growing number of people affected by a chronic condition is a cause of anxiety to public health authorities in terms of health policies and is a concern for healthcare professionals. People with chronic illnesses need to reorganize their lives as their previous ones no longer exist. They are confronted with new information directly linked to specific knowledge of their disease and indirectly to the uncertainty of its outcome [4]. To embrace this, healthcare policies are oriented towards the implementation of means by which people can be encouraged to form a care relationship centred on empowerment and expertise in the narrative of the disease [5]. From a paternalistic approach [6] to a care relationship emphasizing knowledge, decision-making, and the positioning of people, the objective is access to disease management and autonomy [7]. When a person’s environment is favourable, empowerment, defined by Gibson (1991) as a process of increasing someone’s ability to solve their own problems and to mobilize the necessary resources can also be developed [8].

Thus, two elements are important to support those with chronic conditions: a favourable environment and competent professionals whose support is focused on the patient’s needs. The role of healthcare professionals, including advanced practice nurses, is transformed. This review examines the environment of people with chronic disease and the characteristics necessary to develop it into an empowering one.

## Background

Helping a person with a chronic disease to achieve autonomy and commitment are addressed in several concepts and theories: capability, empowerment, therapeutic education, care, and health literacy, in addition to commitment [6]. Developing the idea of an environment [9] favourable for the person, whilst allowing them to increase their competency, seems to be a fundamental step in the process of chronic disease self-management.

Empowerment and an enabling environment appear to be particularly helpful elements in the paradigm shift in the management of chronic disease. The concept of empowerment is described in many disciplines, but in the area of health it centres around health promotion and proposed strategies for the management of chronic diseases [10]. The literature document many definitions of empowerment [8, 11]. Empowerment is a process by which control over a patient’s health is undertaken with the aim of improving their capacity to meet their own needs, and thus to take control of their lives [12]. The ability to become responsible for one’s own life is allied to a complex experience involving health professionals, which self-determination and a person-centred approach to care are fundamental.

In parallel, different analyses of this concept have taken the notion forward in various healthcare settings. At the level of community health, empowerment is defined as a sharing and a willingness to give. Ideas of the therapeutic relationship between the community health nurse and the individual, consensus decision-making, power sharing and focusing on strengths rather than weaknesses are described as attributes of this concept [13]. In the setting of in-hospital critical care, this definition is rather focused on managing challenges and overcoming impotence. A translation of “powerlessness”, it represents a starting point but is the opposite of empowerment. To achieve this, the formation of a partnership between the professional and the person in a conducive environment which includes mutual respect are all essential elements for empowerment [14]. In this way, the development of empowerment is based on the capacities of each individual to make his own decisions when he understands his environment, enabling him to regain an active role in his own life [15]. The concept of an enabling environment was proposed in 2005 in the field of constructive ergonomics where employee health and performance is paramount [16]. This concept is inspired by the idea of defined capabilities as a set of functions specific to humans, allowing them to achieve freedom to make their own life choices [17]. From the preventive point of view, Falzon argued that the characteristics of an enabling environment are found in preserving a person’s capacities of action, which are universal in that they take into account differences (diseases, ageing) and developmental as they contribute to new skills promoting autonomy [18]. Fernagu Oudet [19] focuses not on skills but on abilities and capabilities, for which resources are necessary, and are both internal to the individual and external. Fernagu Oudet presents them as conversion factors: individual (gender, age, experience, level of training), social, organizational, and environmental. These factors help to facilitate the implementation of such an environment, but not necessarily invariably.

An enabling environment is characterized by a set of individual, technical, organizational and social conditions that is not deleterious and which allows the individual to attain a sense of freedom and progress [20]. Thus, it gives agency to a person’s power to act by developing the person’s capacities to exercise this power [16, 19]. The capacity for action would be the result of the empowerment process, but only when the necessary resources are available in the environment.

## Methods

This is an integrative review, allowing a combination of different types of studies and data sources. This methodology follows the rigorous and systematic approach proposed by Whittemore and Knafl [21]. The five recommended phases were followed: identification of the problem, research in the literature, evaluation of the data, analysis, and presentation of the results. This method focuses on the elements of understanding and qualifying an enabling environment for the person with chronic disease [21].

### Search methods

The MEDLINE, CINAHL and Web of Science databases were used and searched from January 1, 2009, to December 31, 2019, to identify articles dealing with chronic disease and its effects on the person and their environment. To identify all the relevant literature, a search using the words “environment” “enabling environment” “patient empowerment” “empowerment” “capabilities approach” “patient chronic illness” was carried out. Two search equations were selected to associate all the important keywords related to the research question.

(Environment) OR (enabling environment) AND (patient empowerment) OR (empowerment) AND (patient chronic illness) and (capabilities approach) AND (patient chronic illness).

The first equation combines the environment, empowerment, and chronic disease, the second adds the notion of capability. Articles were included when they described the concepts of empowerment in the context of chronic disease as well as that of the individual’s environment. No restrictions were applied to the design of the studies.

Studies related to acute disease and surgery were excluded. Articles written with broad notions of empowerment, self-management and self-care and not specifically related to research were not selected. The inclusion and exclusion criteria are shown in Table 1.

**Table 1:** Inclusion and exclusion criteria

Inclusion criteria	Exclusion criteria
-The impact of these interventions on the person in terms of autonomy, involvement, empowerment, power and learning, quality of life in adaptation to the disease are explored in the results -The study population is made up of adults with one or more chronic diseases -The articles report on primary study -Elements comprising the notions of empowerment, capability and an enabling environment are researched and considered as intervention for people in the management of their disease	-Age 18 years or younger -People with pathologies other than chronic diseases -Studies on interventions that do not consider the notion of empowerment or the environment -Studies failing to identify the concepts of autonomy, accountability, support, power, and learning -Studies relating to a literature review

Eligible articles were selected by two independent researchers (VL, JMM) based on the relevance of the titles and abstracts found in the research. This was performed according to the inclusion and exclusion criteria.

This integrative review was guided by Preferred Reporting Items for Systematic Reviews and Meta-Analyses (PRISMA) [22].

### Search outcome

**Fig. 1:**
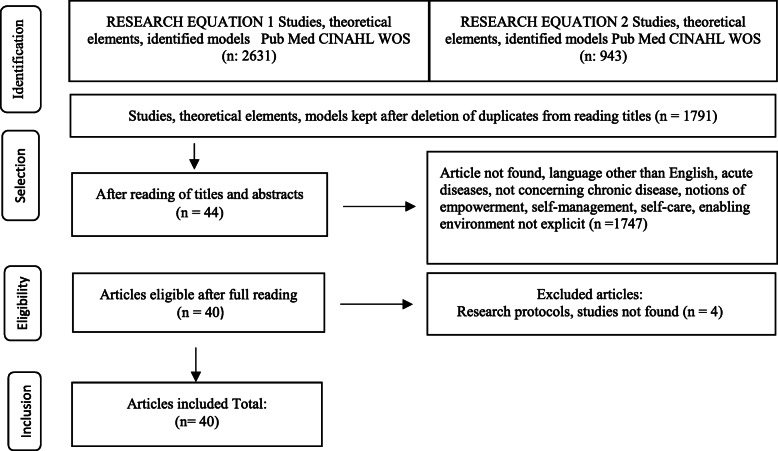
Flow diagram of the article selection process (PRISMA 2020)

A total of 3574 articles were identified on the three databases (Medline, CINAHL and Web of Sciences) using two search equations. After eliminating duplicates with Zotero, and reading the titles, 1791 articles were retained. Assessment of titles and abstracts against the inclusion criteria excluded 1747 articles. The remaining 44 articles were read in full and four were excluded as they pertained to research protocols whose studies were not found (Fig. 1).

**Fig. 2:**
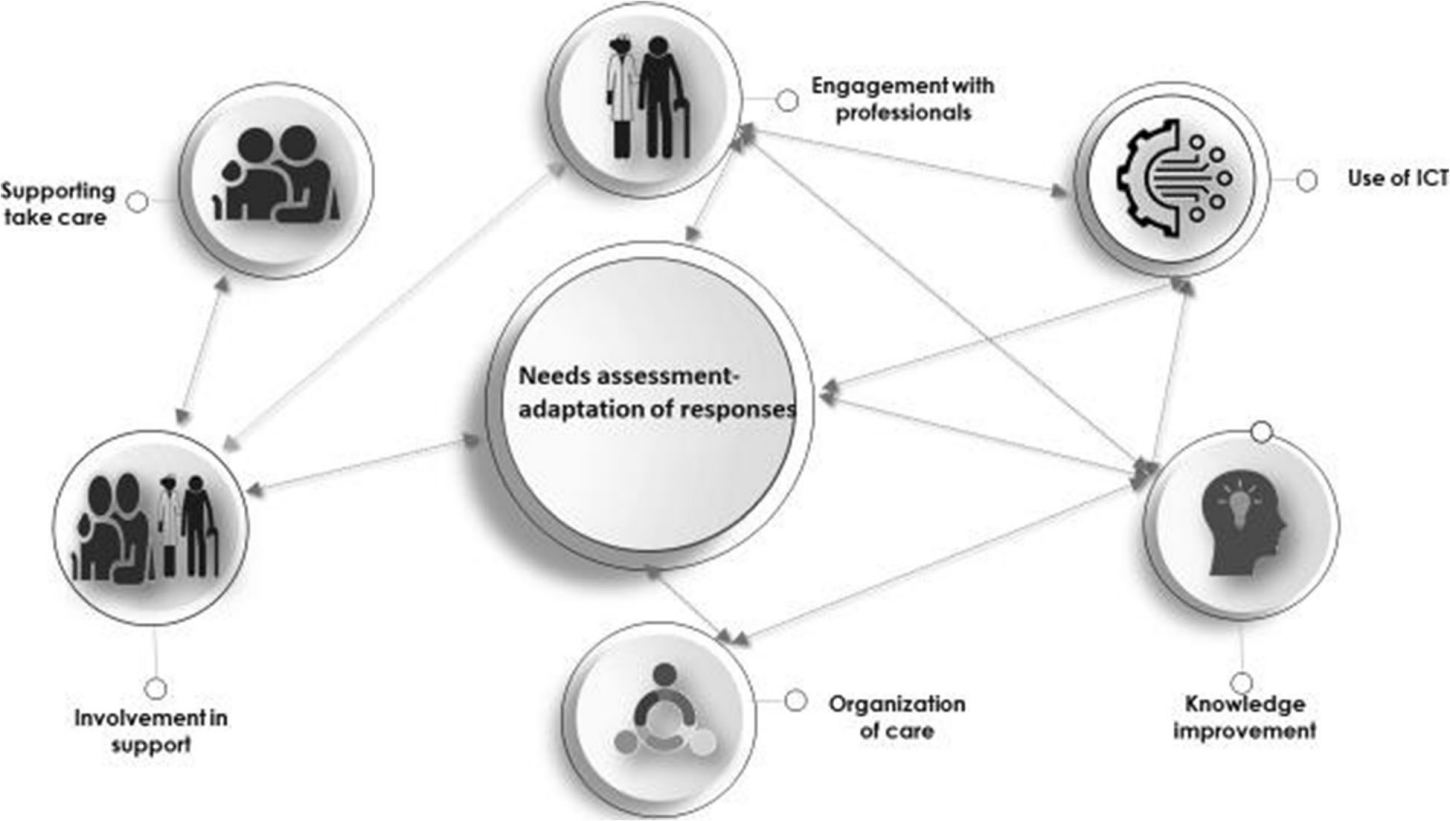
Interacting elements making up an enabling environment

#### Quality appraisal

The methodological quality of the scientific studies selected was verified by the grid “Mixed Methods Appraisal Tools” version 2018 [23]. This tool allows the methodological quality to be assessed for five categories of studies, with the help of the determined criteria. Although it is not advised by Whittemore we performed an overall score calculation as this makes it possible to identify the strengths and weaknesses of each study. However, Whittemore points out that it is difficult to assess the quality of studies and compare them because they use different designs [21].

#### Data extraction

The data were extracted and transcribed in a summary table and verified by the second researcher (JMM). The following data were extracted: author, year of publication, concept, methodology used, objective and main results regarding the enabling environment. The participation of two independent researchers ensures the rigor of data extraction [21].

#### Synthesis

The 40 studies were tabulated according to author, year, type, objective of the article, and the main results concerning the enabling environment (Table 2).

By adopting the Whittemore (2005) recommendations, the different concepts and occurrences have been identified and classified in subgroups. The analysis of recurring themes and the coding of the articles were carried out manually and with the support of the qualitative data analysis software Maxgda 2020. The categorization was carried out according to the number of occurrences and the concepts studied.

## Results

The concept of empowerment is found in half of the articles, that of self-management and self-care appear in the other half. The concept of empowerment has been steadily increasing in the last 10 years and interest in this idea has continued to grow. Most of the articles originate from Europe (*n* = 21), with the other half divided between America (*n* = 8), Asia (*n* = 6) and the Middle East (*n* = 4). Several research designs were used, including quantitative and qualitative studies, focus groups, expert opinions, and congress feedback. Articles made it possible to describe the experiences and practices of professionals and people in the management of their disease and in connection with the development of an enabling environment. Diabetes is the most studied condition (38%), followed by undifferentiated chronic illnesses (36%), cardiovascular diseases, and cancers. The ages of the populations studied depend on the country of origin, with adults over 18 years old being the most common.

### Themes

Living with a chronic disease requires that people adapt, and these articles highlight the elements necessary to them for an environment which qualifies as enabling. Thus, these elements have been classified into seven categories, each of them being determined by components. Each one is described below.

#### Needs assessment-adaptation of responses

In current social usage, the idea of need is difficult to pin down and different for everyone. However, chronic illness forces people to change their behavior and activities in order to move toward physical and mental well-being [32]. This category contains three components: assessment of person-related, illness-related, and emotional needs. Being listened to is essential, especially regarding the concerns of individuals [47]. Shared decision making between the person and health care professionals also seems fundamental [45] as well as support in the health care system [31]. Chronic disease management emphasizes the emotional needs of patients [59], with the need for psychological support frequently mentioned [58].

#### The supporting “take care”

Various forms and modalities of support are cited. However, this often focuses on medical disease management and the behaviours to be adapted (treatments, diets), with little consideration for the management of emotions and consequences of the disease [32]. This category includes three components: emotional, social, and psychosocial support. Patients express a need for psychological support to reduce anxiety and stress related to the different stages of the disease [58]. Individuals need support from professionals to promote self-management [42]. The implementation of a care program based on individualized goals, addressing psychosocial aspects related to the disease, contributes to empowerment [30].

#### Involvement in support

Studies emphasize the central role of support networks. To qualify this category, four components have been highlighted: the centrality of the family, the role of peer helpers, online social networks, and the place of the community. The family must be integrated into the care process [34, 36]. The role of peers [24] is described as facilitative [29] with interactions between people with the same disease [25]. Similarly, it allows for experiential exchanges about the disease [48, 50]. However, younger people prefer online social networks, because they are easier to access than peer interaction [48].

#### Knowledge improvement

Chronic disease management requires the acquisition of essential knowledge [55]. Within this category, two components have been highlighted: support for skill acquisition and different sources of learning. Skill acquisition improves the quality of life of the person with the disease [28], whilst generating a sense of control over it [56, 59, 61]. Patients learn in multiple ways, but professionals remain their primary source of information [57, 58, 62].

#### Engagement with professionals

The disease care process involves both the affected person and their entourage and the health professional, each with an important and continuing role to play. Thus, four components have been identified: the establishment of the relationship, the interaction between the parties, the collaboration between the parties and the person-professional partnership.

A climate of trust that allows people to speak freely and feel comfortable with the professional is undoubtedly one of the conditions for establishing a good relationship. The concept of self-management has led to a reconfiguration of roles [24, 27, 28, 31, 33]. The multi-professional team model is important for monitoring individuals by providing follow-up to address their needs [25, 44, 46].

#### Use of information and communication technologies (ICT)

ICTs are a set of technological tools and resources that allow the transmission, recording, creation, sharing or exchange of information in a different modality to help patients manage their disease. For this, three components have been retained in the studies: utilization of the internet, the role of mobile applications and the use of the telephone. The online support group provides members with shared experiences. Individuals feel that they are caregivers of themselves, rather than passive recipients of care, which allows them to position themselves vis-à-vis professionals by giving their own opinions. The sense of ownership of their illness is also significant [29, 33, 58, 62]. Patient education is the most common functionality found with blood glucose monitoring. In contrast, applications for the psychosocial aspects of the disease are not widely found [35, 45, 51]. The implementation of a telehealth program with telephone use between a professional and a patient has sparked interest in this path to empowerment [60].

#### Organization of care

Understanding the disease but especially integrating it into the patient’s life appears to be essential but is complex given the variability in each person’s approach to understanding their own health status [35, 43, 63]. Within this category, three components have been identified: design of care, criteria for care, and organization of interventions. Encouraging the patient to set their own goals increases motivation, which is important for education and care. Self-care practices are a way of demonstrating that the person’s expertise should be considered in the professionals’ care proposal [39]. Similarly, the level of social support should be pre-assessed by nurses before implementing interventions. The location of care is changing, with a shift from traditional settings such as the health care facility to more informal settings such as the individual’s home. Thus, the services offered, including home visits and telephone follow-up, have a beneficial effect on disease management. They allow direct contact between the professional and the person by taking place in a familiar environment [40]. These real-world interventions allow professionals to visualize the environment [54].

### Interactions

Alongside these seven categories of elements describing an enabling environment, the notion of interaction between the categories was apparent from the selected articles, which showed how synergy seems fundamental in the construction of this environment. Interactions are defined as actions exerted between phenomena or entities, or people influencing each other [64]. As part of this review, several interactions between the components of the enabling environment were highlighted (Table 2).

**Table 2:** Summary of selected articles

Author Year	Type	Purpose of the article	Main results concerning the enabling environment
Angwenyi 2019 [24]	Mixed-method study: interviews, focus group, observations (*n* = 140)	To show that support for Self-management in disadvantaged settings contributes to Empowerment	*Professional/patient interaction: time, supportive environment *Explain prevention approaches, encourage *Home visits appreciated *Learning achieved: 50% through network (religious leader, community and civil society organization, volunteer), radio and television *Peer support *Group problem-solving approaches and a supportive Collective problem-solving approaches and a supportive environment to cope with the stress of the disease
Brady 2017 [25]	Qualitative study: semi-structured interviews (*n* = 21)	Showing the use of the internet as a form of Empowerment	*Access to online health information: Confidence, routine questions related to illness, no questions related to acute side of symptoms. *Allows interaction with health professionals to make decisions *Communicate with others with same disease *Interactions with peers provide support *Supportive reciprocal relationship versus patient passive with specialized care *Online community
Bravo 2015 [12]	Mixed: Scoping review and semi-structured interviews (*n* = 19)	Develop a conceptual map of empowerment including relationships with health literacy, self-management and shared decision making	*Empowerment level is modifiable by care interventions implemented by professionals and the care system *Health education is necessary but not sufficient for empowerment *Self-management: realistic and personally meaningful goals *Partnership between professionals and patients: informed decision making
Chang 2015 [26]	Descriptive study via questionnaire (*n* = 306)	Identify factors of self-care behavior in elderly patients with hypertension	4 predictors of self-care: *empowerment *social support *depression *perceived severity of illness where to evaluate these variables to support people
Chow2014 [27]	Randomized controlled trial (*n* = 312)	To examine the effects of a nurse case management program for older adults with comorbidities at hospital discharge	For effective care interventions must incorporate: *a comprehensive discharge needs assessment *support for patient-centered processes *shared decision making Empowerment interventions increase self-efficacy and decrease hospitalizations Home visits allow interventions in the person’s environment *It is the relationship between the nurse and the patient and not the platform that allows for improvements *Importance of setting up an RPN rather than a generalist because of the different approach
Cojocaru 2014 [28]	Congress	To show the importance of developing Self-Management in people with chronic diseases	*Professionals consider self-management around structured education *For the patient: complex, multifaceted and non-linear process Patient engagement in self-management depends on: disease type, time, gender, age, socioeconomic status, self-efficacy and social support network *Self-management: major issue for positive health outcomes and costs *Importance of the physician-patient relationship configuration
Cooper 2019 [29]	Expert opinion	Show how Self-Management and education can support Empowerment	5 points for self-management: *collaborative care *self-responsibility *focus on individual situations *structured support *liaison with community agencies Importance of time between professional and patient
Cortez 2017 [30]	Randomized controlled trial (*n* = 238)	Evaluating the effectiveness of an Empowerment program for metabolic control in patients with diabetes	*Positive metabolic results after implementation of the empowerment program *program based on individualized objectives: psychosocial, behavioural and clinical aspects
Delaney 2019 [31]	Qualitative phenomenological study (*n* = 15)	Explore and describe the lived experience of chronically ill adults receiving nurse coaching	*Being listened to and heard increases the patient’s power and sense of empowerment *environment of safety, trust, empowerment *examine the patient experience *interaction between professional and patient *need for guidance in the care system
Elissen 2013 [32]	Qualitative study in 13 European countries	To show whether Self-Management support is integrated into care approaches	Self-management: key behaviour in chronic disease for effective management, *similarity between countries: nursing, care setting *difference: mode and format of support *Support activities: medical and behavioural management of patients, less emotional management and consequences of illness *support for self-management focused on individual needs: provider time and resources *nature of patient-physician communication and interprofessional work
Fisher 2017 [33]	Expert opinion	Provide a practical framework for organizing and structuring empowerment programs to improve their use	*building the relationship with the professional * productive interview focused on patients’ needs: competence, autonomy, relationship - patients’ motivations and preferences * respect for patients’ needs
Fotokian 2017 [34]	Qualitative grounded theory study with interviews and field notes (*n* = 24)	Illuminating the Empowerment experiences of patients, their families and caregivers	*importance of families in the management of the disease *cooperation with professionals *various sources of information: internet, radio, peers, discussion with professionals
Hellings 2017 [35]	Expert opinion	Respond to various commissions on prevention and self-management in the context of respiratory diseases	*Mobile application for Education and Self-management *implementation of prevention strategy
Hernandez 2012 [36]	Quantitative study via questionnaires (*n* = 378)	Evaluating the effect of Empowerment on adherence and self-care behavior in Diabetes	*Knowledge is not enough to produce self care * patient adherence *psychosocial support *giving the patient an active and central role in care
Hoffman 2013 [37]	Expert opinion	Describe through examples how nurses apply symptom self-management theory to patients’ perceived self-efficacy	*5 skills for self-management: problem solving, decision making, resource utilization, professional and patient partnership, actions to manage health status * Idea of control, empowerment and confidence
Isaksson. 2015 [38]	Quantitative study via questionnaires (*n* = 159)	Describe the perceptions and associations between Empowerment, Self-Management and support needs in a rural community	*Need for professional support at the beginning of the disease and 15 years after *need for emotional support and family support * cultural influence *notion of visibility of the disease, if invisible difficult to ask for help *purpose of self-management: quality of life and well-being
Johnsen 2017 [39]	Qualitative study via interview (*n* = 16)	Determine how the concept of Empowerment manifests itself in the cancer patient	*Mastery of treatment and care (ability to say no) *knowledge and skills *care system responsive to people’s concerns and needs no clear link between empowerment and self-care (some patients do not want self-care)
Kärner Köhler 2018 [40]	Cross-sectional quantitative study (*n* = 157)	Exploring the relationship between empowerment, self-efficacy and well-being	*Importance of individualized follow-up by focusing on patients’ beliefs, needs and goals *Collaboration between patients and professionals by helping to raise awareness of patient needs, goals and patient’s needs, goals and beliefs *communication between professionals and patients *use of resources to solve problems
Korpershoek 2016 [41]	Qualitative study with semi-structured interviews (n = 15)	Identify and explain the underlying process of self-management behavior during disease aggravation	*Interventions corresponding to patients’ perceptions, abilities, needs and requests for care * 2 skills for self-management (recognition of worsening and taking action)
Kristjansdottir 2018 [42]	Qualitative study via interview (*n* = 39)	Explore patients’ talk about their strengths for their health and well-being	*relation and support of professionals *supporting the forces for self-management and wellbeing. *self-management: priorities, stress reduction, goal setting, knowledge and support *environment for a healthy lifestyle
Magnezi 2014 [43]	Quantitative study via questionnaires (*n* = 296)	Evaluating the effects of participation in an online social health network	*greater impact on younger people (20–29 years) *information role *notion of immediate results, information without waiting for a medical consultation *personal questions are easier to ask on the personal questions are easier to ask on the network than to peers
Musacchio 2011 [44]	Quantitative study using medical record data (*n* = 1004)	Document the impact of an empowerment program on clinical outcomes, including reducing visits to a diabetologist	*the importance of a multi-professional team in the in the care, role in the follow-up *telemedicine (internet and telephone) generates interaction between patient and doctor, immediate response *social network, email, text message: immediate feedback without waiting for the consultation
Nie 2016 [45]	Expert opinion	Examine the characteristics and types of health information in diabetes mobile apps in the context of self-management	*applications are related to education (75%) in diabetes and then blood glucose monitoring, diet and exercise *few applications on psychosocial support *research on applications must take into account cultural aspects
Prigge 2015 [46]	Cross-sectional quantitative study (*n* = 1622)	Document the benefits and best practices that should guide Empowerment	*need for competence versus need for autonomy vary according to the situation, in severe conditions competence is more effective for empowerment * patient-centered medicine, focused on the patient’s needs and fears *strategies for interaction between doctor and patient *reliable and formal knowledge platform
Ramsay 2012 [47]	Qualitative study via interview (n = 29)	To study the understanding, acceptance and use of the concept of Empowerment in a low-income clinic	*empowerment perceived as responsibility *passive role of the patient because professional gives instruction for self-management and patient is responsible for implementation *empowerment refers to “doing what the patient is supposed to do *professionals need to elicit questions, explain choices rather than recommend therapy *need to listen, to have patients’ concerns addressed
Santos 2017 [48]	Quantitative randomized controlled trial (*n* = 238)	Compare adherence and Empowerment for self-care and glycemic control practices in group education and home visit strategies	*Group education and home visits promote change for adherence and empowerment by providing an important environment (notion of time), more effective with the group *allows for the development of caregiving skills such as decision making, autonomy and the experience of living with the disease *Role of peers, exchange of experience
Schildmeuer 2018 [49]	Expert opinion	Conduct a review of the development of an online health tool to empower patients	*social support from the moment of diagnosis *peers patients forum for experiences, (autonomy, skills and relationship) *ehealth: provide functionalities for self-management. *connecting with peers, relatives
Stoilkava Hartmann 2018 [50]	Theory	Present a care model: KALMOD	*holistic approach to optimize self-management *importance of communication *education practice adapted to each patient
Storni 2013 [51]	Case study via observations and interviews (*n* = 14)	Question the design of a self-care technology that supports a large number of patients	*understanding the home environment for the realization of assistive technology *understanding the place and role of each *Glycemic device refers to medical monitoring but does not address the complexity and uncertainty of patients *appropriate the technology by adapting it to patients’ conditions
Suarez Vazquez 2016 [52]	Quantitative study via questionnaires (*n* = 181)	Analyze the Empowerment experience of patients	*Importance of involvement in empowerment - trust in health care professionals *the more serious the illness, the less involved the patient is *climate of trust generates a positive self-perception of empowerment
Sürücü 2018 [53]	Quantitative descriptive cross-sectional study (*n* = 220)	Studying Empowerment, social support as a factor in self-care behaviour	* Perception of social support has an impact on self-care behaviour *feeling empowered allows for self-management of the disease *training related to behavioural approaches and culture
Tang 2010 [54]	Quantitative study of a cohort (*n* = 77)	Measure the impact of an intervention in diabetes management	*Importance of a continuous intervention, centered on the patient, evolving in relation to the environment and in real life conditions *patient choice of behaviour change leads to greater motivation *clinical feedback *newsletter for self-care behaviour
Vadiee 2012 [55]	Expert opinion	Acquire skills with a patient program	*Patient expertise is a central element *Self-care: an element of chronic disease management for the maintenance of optimum health *Self-care: essential basis for preventive and effective measures
Varekamp 2009 [56]	Qualitative exploratory study with interview (*n* = 64)	Exploring Empowerment in Employees with Chronic Illness	*empowerment training: working on work-related issues and seeking solutions in management *importance of communication here to ask for accommodations *different focus: not on their limitations but on their skills as professionals *develop knowledge and skills * focus on needs
Varekamp 2011 [57]	Randomized controlled quantitative study (n = 64)	Evaluate the effect of a program on employee stress and fatigue	* self-efficacy increases if empowerment *interventions focused on employees’ needs
Vosbergen 2013 [58]	Qualitative study via interviews and focus group (*n* = 23)	Examining the patient experience with Self-Management at different stages of coronary artery disease	*Healthcare professionals remain the preferred source of information *notion of time with the health care professional *online self-management service: tailored to needs * need for psychological support to reduce anxiety and stress
Wong 2012 [59]	Quantitative cohort study (*n* = 1141)	Evaluating the effectiveness of an Empowerment program	*importance of time spent on education *chronic disease management: medical, social and emotional needs
Wong 2016 [56]	Quantitative cohort study (*n* = 24,250)	Evaluating the effectiveness of an Empowerment program on the use of hospital services and care	*Program influence: behaviour change, healthy living *program structures education, decreases frequency of care and hospitalization
Zamanzadeh.2016 [60]	Randomized controlled quantitative study (*n* = 66)	Studying the effect of telephone-based distance learning on Empowerment	*Telehealth program provides structured care: improves relationship, removes barriers of location and time *importance of time spent on education *distance education has a positive effect
Zhang 2019 [61]	Prospective quantitative study (*n* = 60)	Evaluating the effect of health education on patients’ quality of life using empowerment theory	*better understanding of the disease allows the patient to develop self-management and quality of life *health education allows the patient to feel in control of his life and his disease *health education is necessary if there is no empowerment in relation to the disease

Many interactions therefore exist between people with chronic disease and the system of care offered within their environment.

### Data synthesis

The elements qualifying an enabling environment are related to the person with a chronic disease and related to the proposed care system. The two must interact to provide a favourable environment for the development and maintenance of the autonomy of the person. These necessary interactions are co-constructed and fit between patient requests and the proposed organization of care. Although they seem obvious, they are among the issues raised in various professional congresses and have been the subject of debate for many years.

These seven categories of elements participating in characterizing an enabling environment for a person with a chronic disease interact with each other as shown in (Fig. 2).

The arrows between the categories symbolize interactions and are taken from the literature review. The articles selected in relation to the interactions were classified according to the level of evidence of the study, which varied between B and C [65]. Each interaction cited was found in an average of seven articles in the integrative review. Table 3 provides an example of the interaction and the literature references. The seven elements that comprise the enabling environment appear as elements that characterize it. In addition to their designation, it is the interactions between them that make it supportive of the person with a chronic disease.

**Table 3:** Interactions in the enabling environment

Identified needs - Different relationship - Professionals	[24, 31]
Person - Physician	[12, 28, 46]
ICT - Person - Professional	[25, 44, 58, 60]
ICT - Skills	[43]
Needs assessment - Accompaniment	[26, 27, 40]
Person - Peer Helpers	[48]
Needs assessment - Organization of care –Improvement of Skills	[30, 32, 33]
Professional Relationship - Person - Social network	[41]
Organization of care - needs assessment	[56]

## Discussion

The international literature on supporting the autonomy of a person with a chronic illness often focuses on empowerment. Empowerment, as a definition or process, has been described by several authors as the basis for a person to live a better life [11], where it is a matter of developing self-determination [66]. However, for an individual to achieve autonomy, the available resources must foster advantageous conditions, i.e., an adapted environment as well as the ability to use this power to build skills, determination, and abilities [67].

This integrative review of the literature synthesizes the components that make up an enabling environment, allowing for the characterization of an adaptive environment recognized as enabling for a person with chronic illness, which allows individuals to develop their power to act [68].

The results of this study show that such an environment is a system composed of a set of elements resulting from the context of both the person’s own life and the organization of care for the individual’ illness and particular situation.

If the assessment of needs and the adaptation of responses appear to be fundamental, [24, 27, 46, 52, 69] the accompaniment, support, improvement of knowledge and use of ICTs must also be considered for the person [25, 63, 70, 71]. At the same time, the engagement of professionals and the organization of care interact with those above, without which the system would not be sufficiently balanced to enable the desired autonomy.

In ergonomic terms, the description of a supportive environment focuses on improving employees’ well-being, developing their skills, and improving their performance, thereby creating an environment that enhances the quality of their work, well beyond the goal of autonomy [16]. The enabling environment is conducive to the development of empowerment which includes the ability to act and considers a person’s individual situation [19].

The results of this review underline the need for a person-health professional partnership, even if this interface seems to exist already in the context of supporting people with chronic disease.

In brief, in the analysis of the concept of partnership [72] suggests specific attributes including sharing of the decision-making process, relationship, patient autonomy, power sharing. The first consequence of implementing the partnership is the development of empowerment [72]. The partnership between the person and the professional should be considered with “multimodal” interventions [40], and there should be rethinking on an individual basis while respecting the roles, skills, and capacities of people with chronic disease according to their expectations and ambitions [53]. These interventions should integrate a person’s needs and demands depending on the organization of care, opportunities for the health professional.

Similarly, the enabling environment has been explored in the health sector as part of the work on health autonomy support [73]. The supportive environment is a factor contributing to the development of individual empowerment and therefore autonomy, where the axes of education and coordination of the professional’s functions are described. A supportive environment is therefore conducive to the development of a person capacity for action.

The articles included in the integrative review highlight some of these elements, particularly with respect to the development of skills and knowledge that contribute to a person’s autonomy. People learn in different ways and from those around them. Similarly, the emergence of technology as an information tool assists a relationship between the person and the professional. The coordinating function of professionals has also been emphasized, particularly as it is focused on the partnership between the patient and the health care professional.

The identification of a favourable environment for a person with a chronic disease thus requires a balance between several elements at the level of the individual, the professional, the organization and technology.

It is the connection and interaction of these categories that defines a supportive environment. Communication between the individual and the professional creates a trusting relationship for shared decision making, the use of technology increases knowledge and skills and creates more confidence in self-managing the disease, online social support networks and telemedicine create a different relationship with the physician, and finally, a support network generates a safe place for the individual.

Beyond these interactions, the need for support is solicited by individuals and is defined as reaching out to someone reaching out to someone who will accompany and go forward with them [74]. From this definition, Maela Paul (2012) retains some important elements to qualify support: a relational dimension expressed around the patients’ request for support and a cooperative dimension integrating peers, networks, and family.

It is probably the partnership between the health professional - such as the advanced practice nurse - and the individual that is central to the development of interventions and care strategies in which each stakeholder will define his or her place according to the specific needs of the person with a chronic disease.

### Strengths and limitations

Although there are many articles on the concepts of empowerment and self-management, there are few on creating a supportive environment for the individual. The concepts chosen, particularly that of empowerment are difficult to demonstrate and make explicit in the daily lives of people in the context of chronic disease management. We decided not to limit the research to a particular disease and to empowerment, thus allowing a broad view of the literature. Therefore, the method chosen to describe a supportive environment permitted the integration of all data from the existing literature (quantitative, qualitative, and mixed studies, expert opinion, theoretical elements, and nursing models), providing a wide range of elements. However, the rather disparate geographic origin of the data likely impacts the results of the studies, particularly at the cultural level, with results varying by country.

This integrative review provides guidance to health care professionals on developing an empowering environment for those under their care.

## Conclusion

The objective of this integrative literature review was to describe the characteristics found in the literature and so allow the qualification of a supportive environment. The literature shows that autonomy support focuses on empowerment, i.e., the development of the person’s power to act. However, to be able to act, the person must encounter favourable conditions. The elements identified concern, on one hand, the care policies centred on autonomy in care, and, on the other hand, the care concepts taught to health students in recent years. In fact, an enabling environment, in the context of chronic disease, is composed of seven categories of elements: needs assessment, caregiving, accompaniment, engagement with professionals, knowledge enhancement, use of ICT and organization of care. This review has highlighted the interaction between the categories whilst consolidating the elements already described in the literature on care proposals and concepts. It underlines the need to take these characteristics into account if we really want to enable people with chronic illnesses to develop their potential for action.

Furthermore, it specifies the essential contours for the development of an environment linked to autonomy and quality of life.

## Data Availability

All data generated or analysed during this study are included in this published article and its supplementary information files.
